# Experiences of violence during the COVID-19 pandemic among people who use drugs in a Canadian setting: a gender-based cross-sectional study

**DOI:** 10.1186/s12889-023-15929-9

**Published:** 2023-05-30

**Authors:** Anmol Swaich, Lindsey Richardson, Zishan Cui, Kora DeBeck, M-J Milloy, Thomas Kerr, Kanna Hayashi

**Affiliations:** 1 British Columbia Centre on Substance Use, Vancouver, BC Canada; 2 Faculty of Health Sciences, Simon Fraser University, Burnaby, BC Canada; 3 Department of Sociology, University of British Columbia, Vancouver, BC Canada; 4 School of Population and Public Health, University of British Columbia, Vancouver, BC Canada; 5 School of Public Policy, Simon Fraser University, Vancouver, BC Canada; 6 Department of Medicine, University of British Columbia, Vancouver, BC Canada

**Keywords:** Physical violence, Sexual violence, COVID 19 pandemic, People who use drugs, Gender differences

## Abstract

**Objectives:**

People who use drugs (PWUD) experience disproportionately high rates of violent victimization. Emerging research has demonstrated that the COVID-19 pandemic has exacerbated violence against some priority populations (e.g., women), however there is limited research examining the impact of the pandemic on the experiences of violence of PWUD.

**Methods:**

Using data collected between July and November 2020 from three prospective cohort studies of PWUD in Vancouver, Canada, we employed multivariable logistic regression stratified by gender to identify factors associated with recent experiences of violence, including the receipt of COVID-19 emergency income support.

**Results:**

In total, 77 (17.3%) of 446 men, and 54 (18.8%) of 288 women experienced violence in the previous six months. Further, 33% of men and 48% of women who experienced violence reported that their experience of violence was intensified since the COVID-19 pandemic began. In the multivariable analyses, sex work (Adjusted Odds Ratio [AOR] = 2.15, 95% confidence interval [CI]: 1.06–4.35) and moderate to severe anxiety or depression (AOR = 3.00, 95% CI: 1.37–6.57) were associated with experiencing violence among women. Among men, drug dealing (AOR = 1.93, 95%CI: 1.10–3.38), street-based income sources (AOR = 1.93, 95%CI: 1.10–3.38), homelessness (AOR = 2.54, 95%CI: 1.40–4.62), and regular employment (AOR = 2.97, 95% CI: 1.75–5.04) were associated with experiencing violence.

**Conclusion:**

Our study results suggest economic conditions and gender were major factors associated with experiencing violence among our sample of PWUD during COVID-19. These findings highlight criminalization of drug use and widespread socioeconomic challenges as barriers to addressing violence among PWUD during periods of crisis.

## Background

The COVID-19 pandemic has had disproportionately adverse health, social, and economic impacts for members of equity-deserving populations whose ability to weather the abrupt and extreme changes associated with the pandemic and pandemic responses is structurally constrained [1]. As a result, a growing body of literature describes COVID-19 as a “syndemic” wherein its emergence aggravated co-occurring health epidemics and led to heightened health disadvantages among populations that were already most at risk [2]. For instance, emerging research has indicated that the COVID-19 pandemic has exacerbated violent victimization among some priority populations, including women, children, and people who use unregulated drugs (PWUD) [3–5]. A recent rapid survey study found that over 29% of 227 PWUD surveyed in Montreal, Canada reported increased frequency of experiencing violence since the beginning of the pandemic [4]. Although there is limited published research describing factors impacting the experiences of violence of PWUD during the COVID-19 pandemic, in other priority populations (e.g., women), researchers have found links between financial insecurity due to the COVID-19 pandemic and a higher risk of physical mistreatment of children, as well as higher likelihood of intimate partner violence [3,5].

Prior to the COVID-19 pandemic, PWUD have been known to experience disproportionately high rates of physical and sexual violence that are related to adverse health impacts, such as reduced use of health services and increased HIV risk [6–10]. While challenges faced by PWUD have commonly been attributed to individual behavioural shortcomings, policies and programs designed to change individual behaviour have failed to effectively reduce drug-related harms [11]. Meanwhile, researchers have shown that even when PWUD form adaptive survival techniques to mitigate risk of violence, social and structural factors prevent them from doing so [12]. McNeil and colleagues [12] found that although PWUD may want to avoid the greater risk of violence in certain areas where both unregulated drug market and social services are concentrated, they are forced to enter those areas to access cheque-cashing services or to purchase drugs.

Several factors have been linked to experiences of violence among PWUD, including: precarious economic conditions (reliance on illegal or informal income sources, including drug dealing and sex work) [8,13]; social norms and conditions (toxic masculinity, past trauma) [8,14]; physical space (concentration of social services in areas with greater risk of violence; and unsafe living conditions, such as homelessness) [6,10]; and government policy (criminalization of drug use and poverty) [8,15]. Past research has also identified gender differences in experiences of violence among PWUD, including in perpetrators of violence [7], types of violent experiences [8], and factors impacting risk of violent victimization [12]. Notably, at the beginning of the COVID-19 pandemic, researchers in Canada rang alarms that many of these circumstances would both heighten PWUD’ vulnerability to COVID-19 transmission and increase overdose risk [16]. Similarly, the notion that COVID-19 has synergistic effects leads to the question of whether COVID-19 impacted the epidemic of violence already facing PWUD.

During the COVID-19 pandemic, a few key changes in public services and supports may have influenced PWUD’s experiences with violence; however, their impacts on the likelihood of violent victimization has not been fully examined in this population. In Canada, recent qualitative research suggests that the COVID-19 pandemic reduced access to services that provide safety from street-based and intimate partner violence for PWUD [17]. In addition to reduced hours and limited physical capacity in public spaces and services, government emergency income supports were introduced to mitigate the negative impacts from the financial shocks of the COVID-19 pandemic in the Canadian province of British Columbia (B.C.), including businesses closures and lay-offs [18]. Depending on eligibility, during the present study period (between July and November 2020), eligible people in BC could access funds through programs including, but not limited to, the Canada Emergency Response Benefit (CERB; $2,000 per month, up to four months), the B.C. Emergency Benefit for Workers ($1000 one-time payment), a $300 monthly top-up to disability and income assistance payments, and a rental subsidy of up to $500 per month (for three months) [18]. Other supports offered included support in paying monthly bills for utilities (e.g. electricity) [18].

The objective of the present study was to conduct an exploratory, gender-stratified analysis of factors associated with experiencing violence (physical or sexual) among PWUD in Vancouver, BC, Canada during the COVID-19 pandemic. The study will examine how socioeconomic conditions of PWUD during the COVID-19 pandemic related to their experiences of violence and provide insight into the role of government responses to the pandemic. To our knowledge, this study is one of the first to conduct a gender-stratified analysis of violent experiences among PWUD during COVID-19, and will add to the growing body of literature examining the synergistic effects of COVID-19.

## Methods

### Study design and participants

The data for the current study were derived from three ongoing prospective cohort studies of PWUD in Vancouver, including the Vancouver Injection Drug Users Study (VIDUS), the At-Risk Youth Study (ARYS), and the AIDS Care Cohort to evaluate Exposure to Survival Services (ACCESS). As described in detail previously, in all three cohorts, participants are recruited through word-of-mouth and community outreach in the Greater Vancouver area [19,20]. Criteria to join each study varies along the following terms: VIDUS requires participants to be aged 18 years or older, HIV-negative and have injected unregulated drugs (including non-prescribed use of prescription drugs) within the month prior to enrolment, ACCESS requires participants to be over the age of 18, living with HIV and have used an unregulated drug other than or in addition to cannabis within the month prior to enrolment, and ARYS requires participants to be between the ages of 14 and 26, street-involved and have used an unregulated drug other than or in addition to cannabis within the month prior to enrolment.

The study protocols of all three cohorts have been harmonized to permit pooled analyses. In brief, the studies use the same interviewer-administered questionnaire and collect blood/urine samples at baseline and semi-annually afterwards. Written informed consent is obtained from participants and each participant receives $40 for completing each study visit. All cohorts have received approvals from the Providence Health Care/the University of British Columbia research ethics board.

Between July and November 2020, due to public health restrictions related to the COVID-19 pandemic, all cohort interviews were completed remotely over the phone and biospecimens were not collected. After the initial contact through telephone, email, and/or social media to invite participants for a follow-up visit, the VIDUS/ACCESS/ARYS participants who were interested in participating in study follow-up but did not have access to a device to complete the remote interview were offered the option to borrow a pre-paid cellphone that they could pick up from the two study offices in Vancouver (one in Downtown Eastside and the other in Downtown South neighbourhood). For the current study, we created an analytic sample restricted to those who completed a phone interview between July and November 2020, reported having used drugs in the past six months and had valid responses for the questions asking about their gender identity and experience of violence in the past six months. The sample was stratified by the participants’ self-identified gender (men vs. women). Initially, we planned to create another category for those who self-identified as transgender or other non-binary gender. However, the low counts (n = 19) presented challenges with maintaining the statistical power and posed the risk of accidental disclosure. Also, because their experiences with violence might differ from men or women, we excluded them from the present analysis instead of including them in either men or women.

### Study measures

The primary outcome of interest for this analysis was having experienced physical or sexual violence (henceforth, violence) in the past six months (yes vs. no). Independent variables included sociodemographic characteristics, sources of income and changes in income and sources, material security factors, access to health and social services, and drug use behaviours. These were chosen based on past research showing associations with greater rates of violence among PWUD or hypothesized relationships identified by Rhodes’ Risk Environment framework [6,8,11−14]. Sociodemographic variables included were: age (per year increase), self-identified ethnicity/ancestry (Black, Indigenous or person of color vs. white). Sources of income and change in income sources included: receipt of any COVID-19 emergency income supports, employment, receipt of other income assistance (such as disability), informal street-based income generation activities such as panhandling or recycling, drug dealing, sex work, other illegal income generation such as theft, and self-reported change in monthly income since COVID-19 (increased vs. decreased vs. not changed). Material security factors considered were: self-reported change in food insecurity since COVID-19 (more often vs. about the same or less often), residence in the Downtown Eastside (a neighbourhood in Vancouver with high rates of systemic marginalization and drug use), and homelessness. Health related variables examined included: avoiding health or social services at any point since the onset of COVID-19, and mental health symptoms as assessed by the PROMIS short form for anxiety and depression (moderate to severe for either anxiety or depression or both vs. none to mild) [21]. And lastly, drug use and treatment related factors considered included: cannabis use (≥daily vs. <daily), unregulated opioid use including heroin, fentanyl or “down”, the local term for unregulated opioids, (≥daily vs. <daily), unregulated stimulant use including cocaine, crack cocaine, or crystal methamphetamine (≥daily vs. <daily), addiction treatment, and stocked up on unregulated drugs during the COVID-19 pandemic. Unless otherwise stated, variables were dichotomized as yes vs. no, and the timeframe of behavioural variables referred to the six months prior to the interview.

### Statistical analyses

All analyses were stratified by gender. First, we used descriptive statistics to examine sample characteristics stratified by the violence victimization for each gender. We used the Pearson’s Chi-Squared test to examine potential differences in the proportion of individuals reporting experiencing violence between genders. Then we used bivariable and multivariable logistic regression to identify factors associated with experiencing violence. We used an *a priori*-defined backward model selection procedure based on examination of Akaike Information Criterion (AIC) to fit a multivariable model [22]. In brief, we constructed a full model including all independent variables of interest that were associated with the outcome in bivariable analyses at p < 0.10. We removed the variable with the largest p-value and built a reduced model. We continued this iterative process until we reached the lowest AIC score [22]. Receipt of social income was excluded from the multivariable modeling procedures because of the highly skewed data.

In a sub-analysis, we also asked participants who experienced violence in the past six months whether their experience of violence changed since the beginning of the COVID-19 pandemic. Participants selected one of the following response options: “Yes, increased/intensified,” “Yes, decreased” or “No.” We used descriptive statistics to show the distributions of these responses in each of the gender-stratified samples. All p-values were two-sided, and all statistical analyses were conducted using the SAS version 9.4 (SAS Institute, Cary, NC).

## Results

In total, 734 individuals were eligible for the present analyses, including 446 (60.8%) men and 288 (39.2%) women. As shown in Tables 1 and 2, the median age was 47 (1st and 3rd quartile: 32–57) years among men and 44 (1st and 3rd quartile: 34–53) years among women. Among men, 293 (65.7%) self-identified as white, 122 (27.4%) as Indigenous and 28 (6.3%) as other persons of colour, while among women, 124 (43.1%) self-identified as white, 154 (53.5%) as Indigenous, and 10 (3.5%) as other persons of colour. Overall, 77 (17.3%) men and 54 (18.8%) women reported experiencing violence in the past six months and there was no significant difference in the proportions reporting violence between the two gender-stratified samples (p = 0.6078).

**Table 1:** Regression analyses of factors associated with experiencing violence among women who use drugs in Vancouver, Canada, July – November 2020 (n = 288)

Characteristic	Experienced violence^a^		Odds Ratio (95% CI)	Adjusted Odds Ratio (95% CI)
	Yes n (%) 55 (18.9%)	No n (%) 236 (81.1%)		
**Age (median, 1st – 3rd Q)**	38 (29–50)	45 (35–53)	0.97 (0.95–1.00)	
**Ethnicity/ancestry**				
Black, Indigenous or other persons of color	35 (64.8)	129 (55.1)	1.50 (0.81–2.77)	
White	19 (35.2)	105 (44.9)		
**COVID-19 emergency income support** ^**a**^				
Yes	42 (80.8)	191 (82.3)	0.90 (0.42–1.94)	
No	10 (19.2)	41 (17.7)		
**Regular employment** ^**a**^				
Yes	23 (42.6)	72 (30.8)	1.67 (0.91–3.06)	
No	31 (57.4)	162 (69.2)		
**Receipt of social income** ^**a**^				
Yes	52 (96.3)	228 (97.4)	0.68 (0.13–3.48)	
No	2 (3.7)	6 (2.6)		
**Drug dealing** ^**a**^				
Yes	22 (40.7)	64 (27.4)	1.83 (0.99–3.38)	
No	32 (59.3)	170 (72.7)		
**Street-based income generation activities** ^**a**^				
Yes	19 (35.2)	58 (24.8)	1.65 (0.88–3.10)	
No	35 (64.8)	176 (75.2)		
**Illegal income generation activities** ^**a**^				
Yes	9 (16.7)	19 (8.1)	2.26 (0.96–5.33)	
No	45 (83.3)	215 (91.9)		
**Sex work** ^**a**^				
Yes	16 (30.2)	36 (15.7)	2.33 (1.17–4.63)	2.15 (1.06–4.35)
No	37 (69.8)	194 (84.4)		
**Changes in monthly income** ^**b**^				
Not changed	21 (39.6)	65 (27.9)		
Increased	21 (39.6)	132 (56.7)	0.49 (0.25–0.97)	
Decreased	11 (20.8)	36 (15.5)	0.95 (0.41–2.18)	
**Changes in food insecurity** ^**b**^				
More often	13 (24.1)	44 (19.0)	1.35 (0.67–2.74)	
About the same/less often	41 (75.9)	188 (81.0)		
**Lived in Downtown Eastside** ^**a**^				
Yes	33 (61.1)	120 (51.3)	1.49 (0.82–2.73)	
No	21 (38.9)	114 (48.7)		
**Homeless** ^**a**^				
Yes	5 (9.3)	30 (12.9)	0.69 (0.25–1.87)	
No	49 (90.7)	203 (87.1)		
**Avoided health or social services** ^**b**^				
Yes	24 (47.1)	77 (33.9)	1.73 (0.94– 3.20)	
No	27 (52.9)	150 (66.1)		
**Stocked up unregulated drugs** ^**b**^				
Yes	18 (35.3)	58 (25.6)	1.59 (0.83–3.04)	
No	33 (64.7)	169 (74.5)		
**Addiction treatment** ^**a**^				
Yes	34 (64.2)	157 (67.7)	0.85 (0.46–1.60)	
No	19 (35.9)	75 (32.3)		
**Cannabis use** ^**a**^				
≥Daily	18 (33.3)	64 (27.4)	1.33 (0.70–2.51)	
<Daily	36 (66.7)	170 (72.7)		
**Unregulated opioid use** ^**a**^				
≥Daily	28 (52.8)	99 (42.5)	1.52 (0.83–2.76)	
<Daily	25 (47.2)	134 (57.5)		
**Unregulated stimulant use** ^**c**^				
≥Daily	23 (43.4)	85 (36.5)	1.33 (0.73–2.45)	
<Daily	30 (56.6)	148 (63.5)		
**Anxiety or Depression**				
Moderate to severe	39 (81.3)	122 (56.2)	3.37 (1.56–7.31)	3.00 (1.37–6.57)
None to mild	9 (18.8)	95 (43.8)		

CI: confidence interval. OR: odds ratio

^a^ denotes behaviours and events in the past six months.

^b^ denotes behaviours and events since the COVID-19 pandemic began

^c^ includes cocaine, crack or crystal methamphetamine

**Table 2:** Regression analyses of factors associated with experiencing violence among men who use drugs in Vancouver, Canada, July – November 2020 (n = 446)

Characteristic	Experienced violence^a^		Odds Ratio (95% CI)	Adjusted Odds Ratio (95% CI)
	Yes n (%) 68 (16.0%)	No n (%) 358 (84.0%)		
**Age (median, 1st – 3rd Q)**	37 (30,50)	49 (33,58)	0.97 (0.95–0.99)	
**Ethnicity/ancestry**				
Black, Indigenous or other persons of color	27 (35.5)	123 (33.5)	1.09 (0.65–1.83)	
White	49 (64.5)	244 (66.5)		
**COVID-19 emergency income support** ^**a**^				
Yes	59 (76.6)	262 (72.2)	1.26 (0.71–2.25)	
No	18 (23.4)	101 (27.8)		
**Regular employment** ^**a**^				
Yes	43 (55.8)	123 (33.4)	2.52 (1.53–4.15)	2.97 (1.75–5.04)
No	34 (44.2)	245 (66.6)		
**Receipt of social income** ^**a**^				
Yes	76 (98.7)	341 (92.4)	6.24 (0.84–46.56)	
No	1 (1.3)	28 (7.6)		
**Drug dealing** ^**a**^				
Yes	29 (37.7)	77 (20.9)	2.29 (1.36–3.87)	1.93 (1.10–3.38)
No	48 (62.3)	292 (79.1)		
**Street-based income generation activities** ^**a**^				
Yes	28 (36.4)	80 (21.7)	2.06 (1.22–3.48)	1.93 (1.10–3.38)
No	49 (63.6)	288 (78.3)		
**Illegal income generation activities** ^**a**^				
Yes	11 (14.7)	25 (6.8)	2.37 (1.11–5.05)	
No	64 (85.3)	344 (93.2)		
**Sex work** ^**a**^				
Yes	4 (5.3)	9 (2.5)	2.22 (0.67–7.42)	
No	71 (94.7)	355 (97.5)		
**Changes in monthly income** ^**b**^				
Not changed	20 (26.3)	131 (35.5)	Ref	
Increased	38 (50.0)	171 (46.3)	1.46 (0.81–2.62)	
Decreased	18 (23.7)	67 (18.2)	1.76 (0.87–3.55)	
**Changes in food insecurity** ^**b**^				
More often	19 (24.7)	64 (17.4)	1.56 (0.86–2.89)	
About the same/less often	58 (75.3)	304 (82.6)	Ref	
**Lived in Downtown Eastside** ^**a**^				
Yes	30 (39.0)	151 (40.9)	0.92 (0.56–1.52)	
No	47 (61.0)	218 (59.1)		
**Homeless** ^**a**^				
Yes	24 (31.2)	52 (14.3)	2.72 (1.54–4.78)	2.54 (1.40–4.62)
No	53 (68.8)	312 (85.7)		
**Avoided health or social services** ^**b**^				
Yes	30 (40.5)	117 (33.4)	1.36 (0.81–2.27)	
No	44 (59.5)	233 (66.6)		
**Stocked up unregulated drugs** ^**b**^				
Yes	19 (25.7)	59 (16.9)	1.70 (0.94–3.08)	
No	55 (74.3)	291 (83.1)		
**Addiction treatment** ^**a**^				
Yes	48 (64.0)	211 (57.5)	1.31 (0.79–2.20)	
No	27 (36.0)	156 (42.5)		
**Cannabis use** ^**a**^				
≥Daily	28 (37.3)	155 (42.1)	0.82 (0.49–1.37)	
<Daily	47 (62.7)	213 (57.9)		
**Unregulated opioid use** ^**a**^				
≥Daily	37 (48.1)	111 (30.2)	2.14 (1.30–3.53)	
<Daily	40 (52.0)	257 (69.8)		
**Unregulated stimulant use** ^**c**^				
≥Daily	28 (36.4)	105 (28.5)	1.43 (0.85–2.40)	
<Daily	49 (63.6)	263 (71.5)		
**Anxiety or Depression**				
Moderate to severe	34 (50.8)	142 (40.7)	1.50 (0.89–2.54)	
None to mild	33 (49.3)	207 (59.3)		

CI: confidence interval. OR: odds ratio

^a^ denotes behaviours and events in the past six months.

^b^ denotes behaviours and events since the COVID-19 pandemic began

^c^ includes cocaine, crack or crystal methamphetamine

The results of the bivariable and multivariable logistic regression analyses in each of the gender-stratified samples are shown in Tables 1 and 2. As shown, in multivariable analyses among women, sex work (adjusted odds ratio [AOR] = 2.15, 95% confidence interval [CI]: 1.06–4.35) and moderate to severe anxiety or depression (AOR = 3.00, 95% CI: 1.37–6.57) were positively associated with experiencing violence.

For men, regular employment (AOR = 2.97, 95% CI: 1.75–5.04), drug dealing (AOR = 1.93, 95% CI: 1.10–3.38), street-based income generation (AOR = 1.93, 95% CI: 1.10–3.38), and homelessness (AOR = 2.54, 95% CI: 1.40–4.62) were positively associated with experiencing violence.

**Fig. 1:**
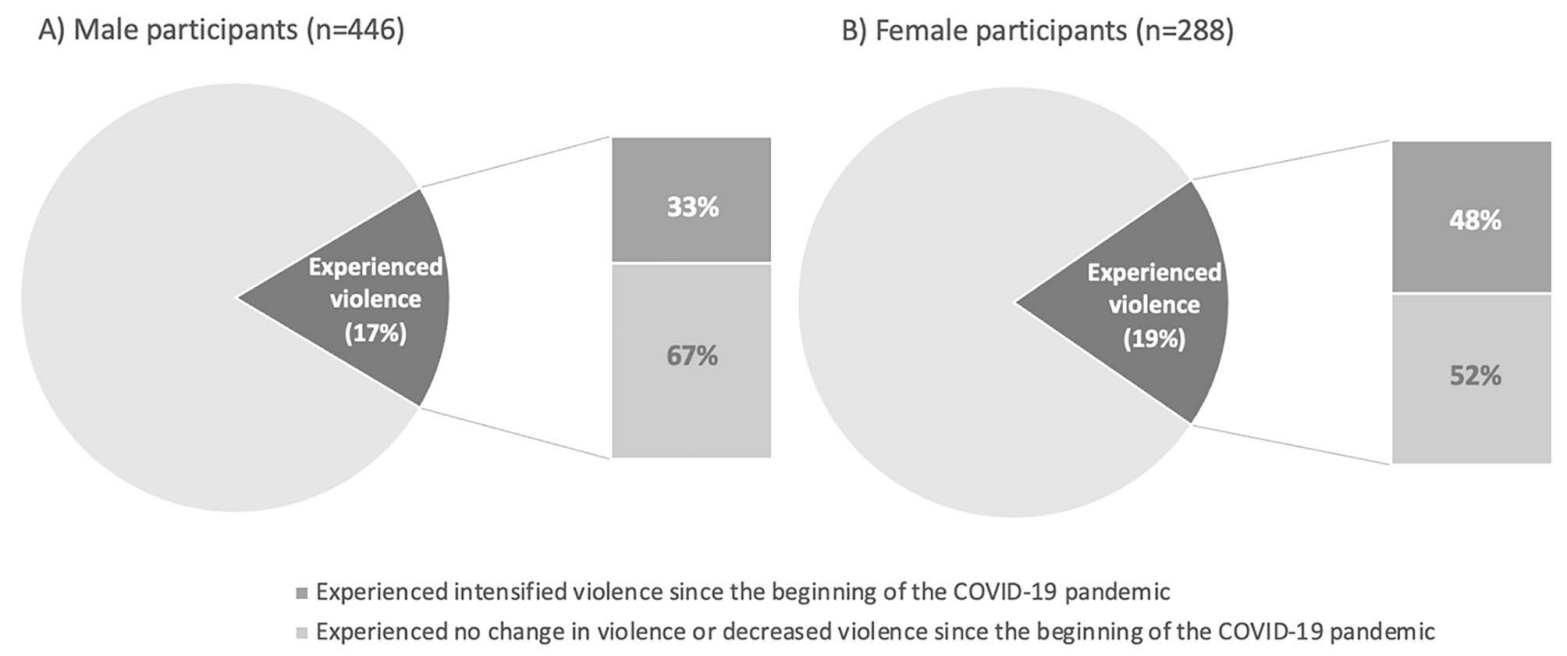
Proportion of participants who experienced an intensification of violence during COVID-19 stratified by gender

The results of the sub-analysis are illustrated in Fig. 1. Among 54 women who experienced violence and answered the relevant question, 26 (48.1%) reported that their experience of violence increased or intensified since the onset of the COVID-19 pandemic, whereas 25 (32.5%) of 77 male participants did so. Further, 2 (3.7%) women and 6 (7.8%) men reported decreased intensity of experiences of violence during the pandemic.

## Discussion

The current study’s findings help us understand the experiences of violence of PWUD in Vancouver during the beginning months of the COVID-19 pandemic. We observed that a large proportion of participants (half of the women and one third of the men) who reported experiencing violence also reported an increase in the intensity of violence since the beginning of the COVID-19 pandemic. This finding is consistent with research showing individuals with a history of experiencing violence were more likely to be victimized during COVID-19 and PWUD experienced increased frequency of violence during COVID-19 [3,4]. Multiple consequences of the pandemic and related restrictions provide plausible explanations for intensification of violence: people may have endured increased exposure to violent relationships due to quarantine or physical distancing requirements [1]; access to support services or opportunities for refuge were curtailed, closed, or shifted online [17]; and heightened stress may have increased violence perpetration [3,5]. Additionally, disruptions in the drug market tend to lead to more violence and COVID-19 related border restrictions may have created new conflict and competition as people tried to purchase from new sources [23,24].

We found gender differences in risk factors for experiencing violence and in reports of experiencing intensification of violence after the COVID-19 pandemic began. Unlike previous research that indicated mental illness and homelessness were associated with violence among both men and women [8], we found anxiety or depression associated with violence among women only and homelessness among men only. While more research is needed, it may be that COVID-19 related factors have amplified the pre-existing patterns of violent victimization that women are more likely to be attacked by someone they know and men are by strangers or the police [8]. This may have led to anxiety or depression for women who were unable to escape from known perpetrators of violence at home or workplace due to physical isolation or reduced access to refuge through social services. Similarly, homelessness may have increased stranger and police interactions among men.

Further, while there was no significant difference in the proportion of women and men in our study who experienced violence (19% and 17%, respectively), a greater percentage of women experienced increased intensity in violence than men (48% and 33%, respectively). Considering that women are likely to hold less power in interpersonal dynamics and street-based economies, such as sex work or drug dealing [12], it follows that COVID-19 related interpersonal, drug market, or street economy conflict may have increased intensity of violence for women who were already at risk of violence.

Our findings reinforce the salience of financial and material circumstances to the risk of violence among PWUD shown in previous research [6,8,13]. We found that sex work was linked to experiencing violence for women, and street-based income generation activities and drug dealing were linked to experiencing violence for men. Although considered significant risk factors for violence among PWUD [13], many PWUD rely on informal income generation methods because they face barriers to regular employment, including stigma, discrimination, criminal records, and abstinence requirements, among others [25]. Further, while researchers have suggested that regular employment may protect against harms among PWUD [25], we found that regular employment was linked to violence among men. This may be because desperate conditions created by COVID-19 related factors, such as financial stress and reduced access to social services, led to increased targeting of those with more resources. However, more research is needed to investigate this.

We did not find a significant relationship between COVID-19 emergency income supports and experiencing violence among men or women. However, it must be noted that we did not disaggregate the different types of support that were available, and individuals who received more income supports through CERB were grouped with people who simply received the income-assistance top-up. Previous research suggests that income assistance rates are not high enough to adequately meet needs of recipients [13,15], and thus, the COVID-19 top-up may not have been enough to reduce participants’ reliance on high-risk income sources or otherwise improve conditions. In addition, people who exclusively depend on informal income generation activities may not have qualified for COVID-19 income supports for people who were laid off work. Future research should examine disaggregated COVID-19 income support data to better understand the impact of different types of income support.

The current study has policy implications for COVID-19 response and suggests that future emergency response measures must integrate consideration of the risk of an intensification of violence among PWUD who are already experiencing violence into service delivery models. Further, our findings that women were more vulnerable to an intensification of violence during the COVID-19 pandemic suggest future policies or programs to address violent victimization among PWUD should reflect gender differences in risk factors and types of violence experienced by men and women [7,8]. This is supported by past research that has found women face safety barriers to some ongoing harm reduction programs [26]. Our recommendations are in line with a recent rapid review that examined the synergistic effects of COVID-19 on gender-based violence, racism, and mental health, and demonstrated a similar need for “gender-responsive” emergency response [2].

This study also highlights the necessity of addressing reliance on informal income sources to prevent violence among PWUD. Researchers have noted the need to decriminalize all aspects of sex work, given that the current criminalization of sex-work clients and third-party coordinators in Canada forces sex workers into dangerous, isolated conditions and reduces their ability to negotiate terms and pre-screen clients [27]. Unsafe labour conditions resulting from criminalization are also a concern in other street-based income generation activities, such as informal recycling which exposes PWUD to police and peer violence despite being considered low-risk [26,28]. Furthermore, offering pharmaceutical alternatives to the unregulated drug supply has been identified as a potential method to reduce reliance on high-risk income sources [29,30]. One study found that PWUD are willing to give up risky income generation strategies if they don’t have to pay for drugs [31]. Ongoing evaluation of these novel interventions through the Assessing Economic Transitions Study will help determine their impact on violence [32].

There are several limitations to the present study and results should be interpreted with caution. The results may not be generalizable to all PWUD in Vancouver because VIDUS, ACCESS, and ARYS are not random samples. In addition, the phone interview requirements may have introduced some selection bias by missing more marginalized cohort participants who did not have access to phones. However, efforts to provide mobile phones to participants who did not have them may have mitigated the effects of this selection bias. Second, because the data were all self-reported there is a possibility that social desirability bias impacted the results due to the stigma attached to the examined topics (e.g., drug use and violence). Similarly, recall bias may have affected results, including in our sub-analysis examining changes in experiences of violence after the COVID-19 pandemic. In addition, interviews were conducted over a five-month period and individual participant responses may reflect different COVID-19-related restrictions and available supports. Also, as 102 (22.9%) men and 92 (31.9%) women were interviewed in July or August 2020, there is a chance that some of their experiences of violence in the past six months may have referred to pre-pandemic periods. Our gender-based analysis of violence is limited because transgender and other non-binary participants were excluded due to low participant counts, a population that is known to experience high rates of violence. Finally, future research should examine disaggregated COVID-19 income support data to better understand the impacts of the different types of income support.

## Conclusion

In summary, we found that approximately a fifth of PWUD in our sample experienced physical or sexual violence during the first few months of the COVID-19 pandemic in Vancouver, B.C., Canada. We found that a large proportion of participants who previously experienced violence reported an intensification of violence during COVID-19. This was true for more women than men, underscoring a need for targeted measures that account for gender differences during emergency response, and more broadly, to address violence in this population. The current study highlights that reducing reliance on high-risk income sources and improving workplace safety may be key to reduce exposure to violence among PWUD. This study adds to previous literature that has identified the critical need to address economic conditions and dangerous environmental circumstances created by criminalization of drug use that expose PWUD to violence.

## Data Availability

The de-identified datasets used and analysed for the present study may be available from the corresponding author upon reasonable request and permission from all the research ethics boards involved in this study.

## List of abbreviations

ACCESS: AIDS Care Cohort to evaluate Exposure to Survival Services
ARYS: At-Risk Youth Study
AIC: Akaike Information Criterion
B.C.: British Columbia
CERB: Canada Emergency Response Benefit
PWUD: People who use drugs
VIDUS: Vancouver Injection Drug Users Study
